# Risk prediction model based on blood biomarkers for predicting moderate to severe endoscopic activity in patients with ulcerative colitis

**DOI:** 10.3389/fmed.2023.1101237

**Published:** 2023-02-21

**Authors:** Xiaojuan Li, Zihui Tang, Yanbing Liu, Xinyan Zhu, Fei Liu

**Affiliations:** ^1^Department of Gastroenterology, Minhang Hospital, Fudan University, Shanghai, China; ^2^Department of Gastroenterology, Shanghai East Hospital, Medicine School of Tongji University, Shanghai, China; ^3^Department of Gastroenterology, Ji’an Hospital, Shanghai East Hospital, Ji’an, Jiangxi, China

**Keywords:** dynamic nomogram, Lasso regression, ulcerative colitis, endoscopic activity, blood biomarker

## Abstract

**Object:**

We explored developing an internal validation model to predict the moderate to severe endoscopic activity of ulcerative colitis (UC) patients based on non-invasive or minimally-invasive parameters.

**Methods:**

Ulcerative Colitis Endoscopic Index of Severity (UCEIS) and Mayo endoscopic subscore were performed for UC patients who met the criteria from January 2017 to August 2021 through the electronic database of our center. Logistic regression and a least absolute shrinkage and selection operator (Lasso) regression model were performed to screen the risk factors of moderate to severe UC activity. The nomogram was established subsequently. Discrimination of the model was evaluated using the concordance index (c-index), and the calibration plot and 1,000 Bootstrap were used to evaluate the model’s performance and conduct internal validation.

**Results:**

Sixty-five UC patients were included in this study. According to UCEIS criteria,45 patients were moderate to severe endoscopic activity. 26 potential predictors of UC were analyzed by logistic and Lasso regression showed that vitamin D (Vit D), albumin (ALB), prealbumin (PAB), and fibrinogen (Fbg) were the best predictors of moderate to severe endoscopic activity of UC. We used these 4 variables to develop a dynamic nomogram prediction model. The c-index was 0.860, which means good discrimination. The calibration plot and Bootstrap analysis showed that the prediction model accurately distinguished the moderate to severe endoscopic activity in UC patients. The prediction model was verified using a cohort of UC patients with moderate to severe activity defined by the Mayo endoscopic subscore, and it was found that the model still had good discrimination and calibration (c-index = 0.891).

**Conclusion:**

The model containing Vit D, ALB, PAB, and Fbg was a good tool for evaluating UC activity. The model is simple, accessible, and user-friendly, which has broad application prospects in clinical practice.

## Introduction

Ulcerative colitis (UC) is a chronic idiopathic mucosal inflammation of the colon that is usually initialed in the rectum and extends continuously toward the proximal colon, part or the entire colon (1).

Colonoscopy plays a critical role in diagnosing and assessing the severity of UC. The most commonly used scoring systems are the Ulcerative Colitis Endoscopic Index of Severity (UCEIS) and Mayo endoscopic subscore. By grading, the activity of UC can be evaluated accurately. However, endoscopy has some disadvantages as a more invasive procedure, such as the need to take laxatives or enemas for bowel preparation, special equipment and trained endoscopists’ requirements. Besides, colonoscopy has some certain risks in performing with a perforation incidence of 0.005–0.085% and a bleeding incidence of 0.001–0.687%. Elderly patients and patients with inflammatory bowel disease have even higher risks (2).

Many non-invasive or minimally-invasive parameters were associated with inflammatory bowel disease activity or severity in recent years, including clinical features such as stool frequency and hematochezia (3); blood indicators such as C-reactive protein, erythrocyte sedimentation rate, serum amyloid A, ferritin transferrin, haptoglobin, vitamin D (Vit D), albumin (ALB), fibrinogen (Fbg), white blood cell count, platelet count, and cytokines; fecal indicators such as fecal occult blood, fecal immunochemical test, calprotectin, and lactoferrin (4–7). Fecal calprotectin is a non-invasive biomarker that assesses intestinal inflammation and is a more sensitive marker than CRP for detecting inflammatory bowel disease activity (8).

However, with many of these parameters, there is still a lack of a good and reliable tool established by the non-invasive or minimally-invasive parameters to distinguish the moderate to severe endoscopic activity of UC. Therefore, developing the tool for evaluating UC activity by the whole or part of the non-invasive or minimally-invasive parameters panel is very important in current clinical practice.

Nomogram is a graphical tool to simplify the statistical prediction model with simple graphics to generate the digital probability of clinical events, characterized by individualization, user-friendly interface and wide network availability (9). Nomogram has been widely used in clinical prediction model construction. Our study aimed to develop an individualized risk prediction tool for moderate to severe endoscopic activity in UC patients. Based on clinically available non-invasive or minimally-invasive parameters, such as general demographic and clinical characteristics and blood and stool biomarkers, we calculated and picked out the appropriate predictors for recognizing the moderate to severe endoscopic activity of UC, then developed a practical and user-friendly dynamic nomogram. It can be used to evaluate the risk of moderate to severe endoscopic activity in UC patients. We hope the model will help reduce the unnecessary invasive colonoscopy and reduce patients’ pain and potential risks. Nevertheless, it is expected to assist clinicians and patients in following up and guiding UC patients’ self-management.

## Materials and methods

### Study design and patients

This study was a single-center retrospective study. The patients with UC who were hospitalized from January 2017 to August 2021 were searched through the electronic medical database of Shanghai East Hospital, Medicine School of Tongji University. The search keywords were admission or discharge diagnosis of “ulcerative colitis.” The inclusion criteria included the diagnosis was ulcerative colitis based on clinical consensus (10); aged between 16 and 75 years old; records of colonoscopy; comprehensive blood and stool examinations within 1 week before or after colonoscopy, including biomarkers such as blood cell counts, protein nutrition, vitamins. Exclusion criteria included: ulcerative colitis was excluded by follow-up; age <16 or >75 years old; UCEIS could not be performed (e.g., bowel resection or incomplete bowel examination); serious complications, malignant tumor, hematological diseases, serious infection, or impaired liver and kidney function; Vit D supplementation in the past 6 months; missing clinical data (such as patients were missing 26 potential predictors described below). Referring to demographic and clinical characteristics, blood and stool biomarkers, and colonoscopy information of patients to screened eligible patients, 203 UC patients were preliminarily screened, of which 65 UC patients were eventually included in the study (Supplementary Figure 1). According to the inclusion and exclusion criteria and UCEIS scoring standard of the clinical guideline (11), 45 patients with moderate to severe activity and 20 patients with remission or mild activity.

### Endoscopic scores

The endoscopic photographs and descriptions were reviewed through the endoscopic image reporting system, and UCEIS (12) and Mayo endoscopic subscore (13) were scored retrospectively by experienced endoscopists, including vascular pattern, bleeding, and erosions ulcers. Remission (UCEIS 0; Mayo 0); Mild (UCEIS 1–3; Mayo 1); Moderate (UCEIS 4–6; Mayo 2); Severe (UCEIS 7–8; Mayo 3) were defined according to clinical guideline (11).

### Clinical data collection

The potential parameters were considered based on accessibility and clinical simplicity. Data of UC patients were collected from the electronic medical database of our center, including general demographic and clinical characteristics: sex, age (year), body mass index (kg/m^2^), age of onset (year), fever (temperature ≥37.3°C), abdominal pain and hematochezia, stool frequency, complications or comorbidities; Blood biomarkers: Vit D (ng/ml), folate (ng/ml), vitamin B12 (pg/ml), ALB (g/L) prealbumin (PAB, mg/L), retinol-binding protein (mg/L), ferritin (ng/mL), Fbg (g/L), white blood cell count (*10^9^/L), neutrophil count (*10^9^/L), monocyte count (*10^9^/L), lymphocyte count (*10^9^/L), platelet count (*10^9^/L), hemoglobin (g/L) and erythrocyte sedimentation rate (mm/H), and fecal occult blood test. All predictors were collected within 1 week before or after colonoscopy.

### Ethics

This study was reviewed and approved by the Ethics Committee of Shanghai East Hospital, Medicine School of Tongji University.

### Statistical analysis

Categorical variables were described as frequency and percentage, and comparisons between groups were used Chi-square tests or Fisher’s exact tests. Continuous variables were tested for normality by Kolmogorov–Smirnov tests, mean and standard deviation (SD) was used for normally distributed variables, and independent samples *t*-test was used for comparison. For not normally distributed variables, median and interquartile range (*IQR*) were used, compared using the Mann–Whitney U tests. Potential predictors were sequentially screened by univariate logistic analysis and were presented as odds ratio (OR) and 95% confidence interval (CI). Statistically, significant variables in the results were included in the least absolute shrinkage and selection operator (Lasso) regression model, which could reduce the variable coefficient value to 0, thus achieving variable screening. Multicollinearity between predictors may lead to spurious associations and unreliable effect estimates. We used the Lasso regression model to select variables, and Lasso regression limits coefficients by adding regularization terms, to avoid multicollinearity and model overfitting and improve the generalization ability of the model.

The fitting degree of the model was evaluated by the Hosmer–Lemeshow test. Then, the nomogram was analyzed by the “RMS” library and “DynNom” library of R packages. The performance of the prediction model was evaluated by discrimination and calibration. The discrimination was evaluated by the concordance index (c-index), which can be measured by the area under the receiver operating characteristic curve (AUC). The closer the AUC value is to 0.5, the poorer the consistency is. Conversely, the closer the AUC value is to 1.0, the better the consistency, indicating that the predicted probability had high consistent with the actual frequency. Then, the calibration was evaluated by the visual calibration plot. The internal validation was realized by Bootstrap of 1,000 times re-sampling to evaluate the consistency of the predicted probability of the model. Two-tail *p* < 0.05 was considered statistically significant. Statistical analyses were performed by SPSS (version 20.0) and R software (version 4.1.0).

## Results

### Vitamin D, albumin, prealbumin, and fibrinogen were the optimal predictors of moderate to severe endoscopic activity of UC

**FIGURE 1:**
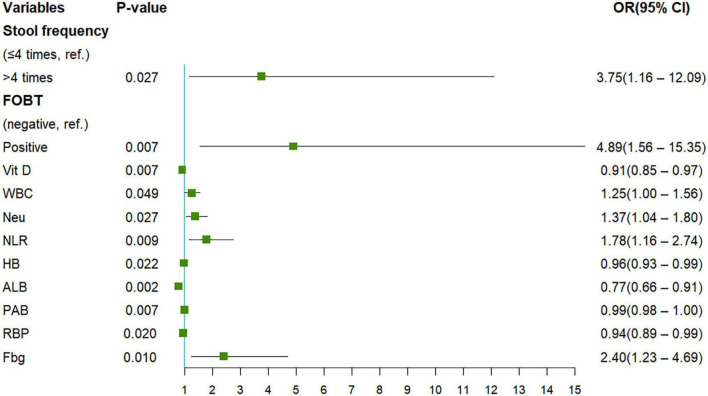
The results of univariate Logistic regression analysis were presented. FOBT, fecal occult blood test; Vit D, vitamin D; WBC, white blood cell count; Neu, neutrophil count; NLR, neutrophil to lymphocyte ratio; HB, hemoglobin; ALB, albumin; PAB, prealbumin; RBP, retinol-binding protein; Fbg: fibrinogen.

**FIGURE 2:**
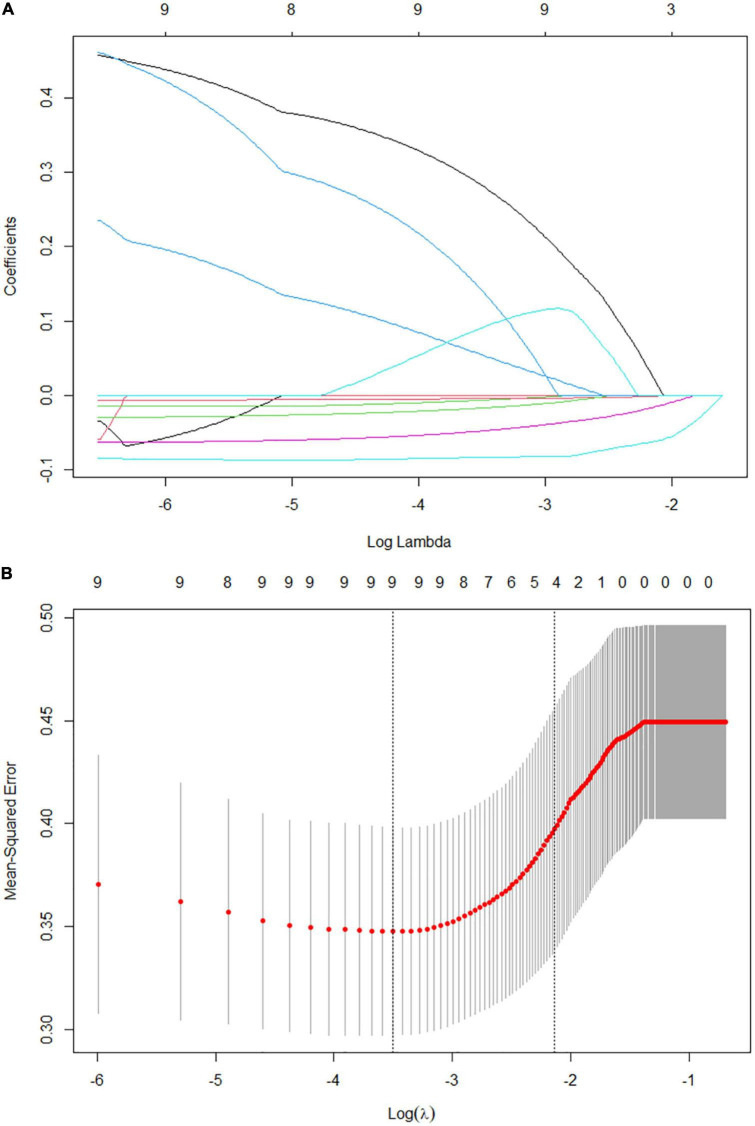
Factors selection for the predictive model by Lasso regression model. **(A)** Lasso coefficient profile plot. As the lambda value increases, the compression parameter increases and the absolute value of the coefficient decreases. The number at the top of the figure is the number of variables. **(B)** The optimal lambda value in the Lasso model was determined by three-fold cross-validation and minimum criterion. The two vertical lines represent lambda values corresponding to the minimum mean square error (left) and one standard error of the minimum mean square error (right). The position of the vertical line on the right is log(λ) = −2.09, corresponding to 4 variables.

The present study included 65 eligible UC patients, 45 of whom had moderate to severe endoscopic activtity according to the UCEIS scoring criteria. The demographics and clinical characteristics are shown in Table 1. We performed univariate logistic regression analysis with 26 potential predictors to evaluate the risk of each variable in moderate to severe endoscopic activity of UC patients. 11 variables with statistical significance were initially screened (Figure 1), including stool frequency, fecal occult blood test, Vit D, white blood cell count, neutrophil count, neutrophil and lymphocyte ratio, hemoglobin, ALB, PAB, retinol-binding protein, and Fbg. Then, the 11 variables were analyzed by the Lasso regression model for further screening. The variations of variable coefficient and the internal three-fold cross-validation of the Lasso regression model were shown in Figure 2. The optimal model was established when log(λ) = −2.09, that was λ equaled the internal cross-validation error reaching one standard error of the minimum mean square error (14). The final screening results showed Vit D, ALB, PAB, and Fbg were the left variates in the model.

**TABLE 1:** The demographics and clinical features of patients with ulcerative colitis.

	Remission or mild activity (*n* = 20)	Moderate to severe activity (*n* = 45)	*p*-Value
Sex (Male/female), *n*	7/13	22/23	0.298
Age, mean (*SD*)	50.15 ± 15.06	50.71 ± 15.21	0.891
≤65 years, *n* (%)	16/20(80)	37/45(82.2)	1.000
>65 years, *n* (%)	4/20(20)	8/45(17.8)	
Onset age, median (IQR)	46.50(32.00-57.50)	47.00(31.00-58.50)	0.966
BMI, mean (SD)	23.55 ± 3.56	22.72 ± 2.71	0.306
Primary, *n* (%)	8/20(40.0)	19/45(42.2)	0.876
Chronic recurrent, *n* (%)	12/20(60.0)	26/45(57.8)	
Fever, *n* (%)	2/20(10)	4/45(8.9)	1.000
Abdominal pain, *n* (%)	6/20(30.0)	14/45(31.1)	0.929
Hematochezia, *n* (%)	12/20(60)	36/45(80)	0.090
Stool frequency, *n* (%)			0.023
≤4 times	15/20(75.0)	20/45(44.4)	
>4 times	5/20(25)	25/45(55.6)	
FOBT, *n* (%)			0.005
Positive	9/20(45.0)	36/45(80.0)	
Negative	11/20(55.0)	9/45(20.0)	
Vit D, mean (SD)	19.37 ± 12.79	11.85 ± 5.95	0.020
Folate, median (IQR)	8.85(7.30–9.32)	7.30(3.85–9.74)	0.211
Vit B12, median (IQR)	375.04(116.50–475.50)	367.00(105.00–545.00)	0.870
ALB, median (IQR)	44.20 ± 3.76	39.18 ± 5.28	<0.001
PAB, mean (SD)	238.12 ± 45.34	188.11 ± 66.10	0.003
RBP, median (IQR)	33.50(29.68–43.50)	27.00(22.20–37.00)	0.015
Ferrin, median (IQR)	95.77(38.30–172.25)	159.00(59.66–96.50)	0.126
Fbg, median (IQR)	3.07(2.60–3.65)	3.57(3.15–4.74)	0.005
WBC, median (IQR)	5.81(4.97–7.50)	7.05(5.31–10.55)	0.046
Neu, median (IQR)	3.57(2.48–4.65)	4.51(3.47–7.14)	0.013
Mon, median (IQR)	0.48(0.41–0.52)	0.55(0.39–0.97)	0.125
Lym, median (IQR)	1.74(1.39–2.08)	1.50(1.08–1.75)	0.047
NLR, median (IQR)	2.17(1.55–2.72)	3.56(2.25–5.35)	0.002
PLT, median (IQR)	258.50(209.25–81.50)	255.00(214.00–4.50)	0.522
HB, median (IQR)	132.50(121.50–48.00)	119.00(109.00–7.50)	0.021
ESR, median (IQR)	15.00(5.25–22.06)	19.00(8.50–34.50)	0.283

Data are presented as the mean ± standard deviation (SD) or median and interquartile range (IQR), depending on whether the variables were normally distributed or not. BMI, body mass index; FOBT, fecal occult blood test; Vit D, vitamin D; Vit B12, vitamin B12; ALB, albumin; PAB, prealbumin; RBP, retinol-binding protein; Fbg, fibrinogen; WBC, white blood cell count; Neu, neutrophil count; Mon, monocyte count; Lym, lymphocyte count; NLR, neutrophil to lymphocyte ratio; PLT, platelet count; HB, hemoglobin; ESR, erythrocyte sedimentation rate. *p*-Value, differences between remission or mild activity and moderate to severe activity in patients with ulcerative colitis.

### Prediction model construction

**FIGURE 3:**
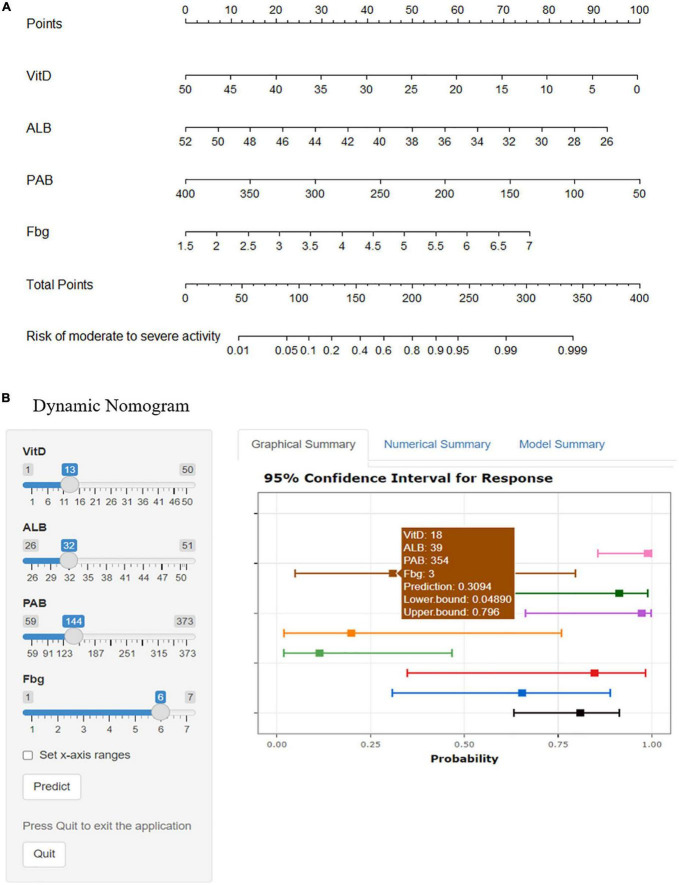
Nomograms of predicting the ulcerative colitis individual risk of moderate to severe endoscopic activity. Vit D, vitamin D, ng/mL; ALB, albumin, g/L; PAB, prealbumin, mg/L; Fbg, fibrinogen, g/L. **(A)** The static nomogram. **(B)** The dynamic nomogram. Select the number on the left and click the Predict button to display the predicted probability and 95%CI of the risk of moderate to severe endoscopic activity for UC individuals in the “Graphical Summary” box on the right. The “Numerical Summary” box displays the specific values and the “Model Summary” box displays the corresponding operators. https://mydynnom.shinyapps.io/vitD_Liu/.

The final model took the 4 variates (Vit D, ALB, PAB, and Fbg) as predictors when log(λ) = −2.09. R software was used to construct a nomogram based on multivariate logistic regression to predict the risk of the moderate to severe endoscopic activity of UC patients by percentage (Figure 3). We showed the model in 2 forms: the static nomogram and the dynamic nomogram (online version). The static graph nomogram does not need the network (Figure 3A). By reading the figure on the bottom axis, the risk of moderate to severe activity in UC patients can be estimated. The detailed manipulation steps are as follows: draw a vertical line from the value of each variate line, read the figure on the “Point Line”; sum up the 4 figures on the “Point Line” to obtain the total figure, read the figure on the “Total Points Line”; then, draw a vertical line from the “Total Points Line,” read the figure on the bottom line, which shows the risk percentage of predicting the moderate to severe endoscopic activity. At the same time, the dynamic nomogram is easier to apply relatively. It has been established on the website^1^ (Figure 3B) with a more concise digital interface. The dynamic nomogram is user-friendly, simple and accurate, showing the individual’s risk of moderate to severe activity with 95%CI through the 4 variable values from each patient.

### The performance and internal validation of the prediction model

**FIGURE 4:**
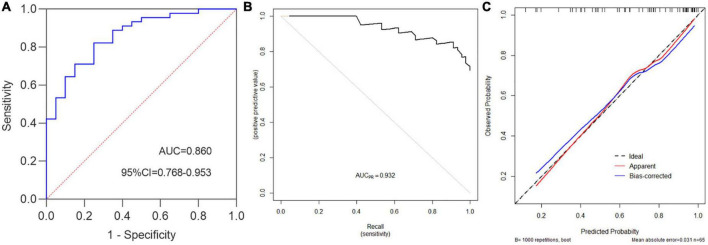
Ulcerative colitis endoscopic index of severity standard was used to evaluate the performance of the prediction model. **(A)** c-index = 0.860, namely the AUC, 95%CI = 0.768–0.953, Youden index = 0.572, sensitivity = 0.822, specificity = 0.750, accuracy = 0.8, positive predictive value (PPV) = 0.881, negative predictive value (NPV) = 0.652, false positive rate (FPR) = 0.250, positive likelihood ratio(+LR) = 3.289, −LR = 0.237, precision = 0.881, recall = 0.822. **(B)** PR curve (Precision vs. Recall), AUC_PR_ = 0.932. **(C)** Prediction model calibration plot, “Ideal” is the ideal curve, “Apparent” is the actual curve, and “Bias-corrected” is the curve corrected by the Bootstrap with 1,000 times re-sampling, the mean absolute error is 0.031.

Hosmer–Lemeshow test was used to evaluate the fitness of the model and UCEIS. The results were *χ^2^* = 3.747, *p* = 0.808, suggesting that the model had good forecasting accuracy. Model discrimination referred to the ability to distinguish patients of different categories, reflecting the predictive ability of the model. The c-index of this model was 0.860 (95%CI 0.768–0.953), indicating that the model could predict moderate to severe endoscopic activity with 86.0% confidence (Figure 4A). The accuracy, precision, and recall were 0.800, 0.881, and 0.822, respectively. Then we drew a precision and recall (PR) curve, and the AUC_PR_ = 0.932 (Figure 4B). 1,000 times Bootstrap analyses were performed for model calibration and internal validation. The results showed a good correlation between UC patients’ actual observed and predicted moderate to severe endoscopic activity, with a mean absolute error of 0.031 (Figure 4C).

**FIGURE 5:**
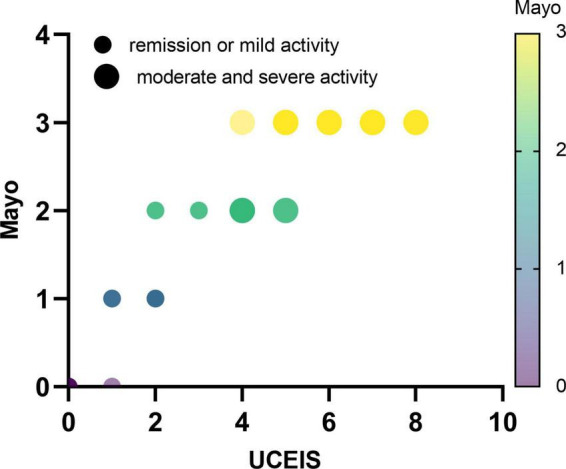
Distribution characteristics of ulcerative colitis endoscopic index of severity and Mayo endoscopic subscore in all UC patients.

**FIGURE 6:**
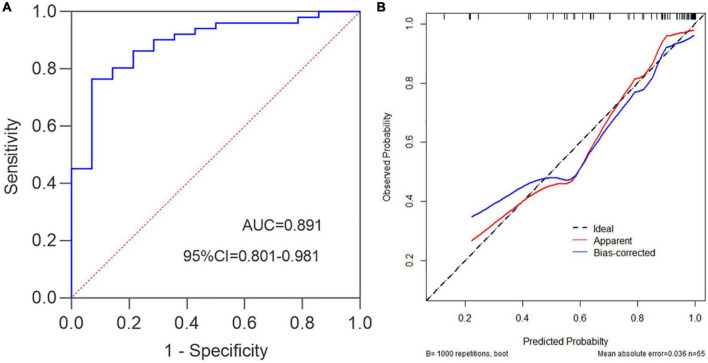
Mayo endoscopic subscore standard was used to evaluate the performance of the prediction model. **(A)** c-index = 0.891, namely the AUC, 95%CI = 0.801–0.980, Youden index = 0.693, sensitivity = 0.765, specificity = 0.929. **(B)** Prediction model calibration plot, the mean absolute error is 0.036.

Subsequently, we further explored the correlations between this model and Mayo endoscopic subscore. The characteristic distribution of UCEIS and Mayo scores in 65 UC patients was shown in Figure 5. Among the 20 UC patients whose UCEIS score was assessed as remission or mild activity, 6 patients were evaluated as moderate activity by Mayo score. 45 patients were assessed as moderate to severe activity by UCEIS and Mayo score. We used the model to evaluate 51 UC patients with moderate to severe activity accessed by the Mayo endoscopic subscore standard. The results showed that the c-index is 0.891 (95%CI 0.801–0.980), indicating that the model could accurately identify UC patients with moderate to severe activity by the Mayo score as well (Figure 6A). Further model calibration also found that the prediction model could better predict moderate to severe UC patients based on Mayo score, with a mean absolute error of 0.036 (Figure 6B). The results indicated that whether UCEIS or Mayo score is used, the model can accurately and easily predict the risk of moderate to severe endoscopic activity in UC patients.

## Discussion

A simple prediction model for moderate to severe endoscopic activity of UC is still lacking based on readily available minimally-invasive blood biomarkers. Previous studies focused on the correlations between single or multiple variables and UC activity or severity, lacking a systematic evaluation model. Based on clinically accessible minimally-invasive parameters, we developed a simple, accessible, practical and user-friendly prediction tool with dynamic nomogram through variate screening by Lasso regression. It is useful to evaluate the risk of moderate to severe endoscopic activity of UC and stratify the disease risk.

We applied to blood and stool biomarkers for variable screening through Lasso regression to construct a model for predicting the individual risk of moderate to severe endoscopic activity in UC patients based on four minimally-invasive parameters. This model can reliably predict the risk of moderate to severe endoscopic activity in UC patients through internal validation.

Mayo endoscopic subscore is based on erythema, vascular texture, brittleness, erosion, ulcers, and spontaneous bleeding. It does not distinguish the depth of ulcers, which all score 3. Ulcers tend to become smaller and shallower during mucosal healing, but Mayo score cannot determine the subtle alternation. Compared with Mayo score, UCEIS can distinguish the depth of ulcers, accurately reflect the actual endoscopic manifestations of patients with severe UC, and more accurately reflect clinical outcomes and short- and long-term clinical outcomes (15). Therefore, this study constructed a clinical prediction model based on the UCEIS, which was also consistent Mayo score. The accuracy of this model in distinguishing moderate to severe activities in UCEIS and Mayo scores was 86.0 and 89.1%, respectively. The internal validations were good with an extremely low mean absolute error (0.031–0.036).

In 2021, Zhang (3) developed a model to predict moderate to severe activity under Mayo endoscopic subscore in UC patients based on fecal frequency, hematochezia, erythrocyte sedimentation rate and intestinal wall blood flow index through transabdominal bowel ultrasound. The model relied on ultrasonographic equipment and experienced radiologists. Another issue was that the study only adopted logistic regression to screen variables without considering collinearity. We used the Lasso regression analysis to avoid the collinearity problems. In addition, the parameters we used were all minimally-invasive biomarkers, which were simpler, more accessible and more economical than bowel ultrasounds.

Vitamin D is a fat-soluble vitamin, 1,25(OH)_2_D is its active metabolic form, and the level of 25(OH)D in the blood can better reflect the state of Vit D in the body. Vit D induces the expression of β-defensin 2 in monocytes and macrophages, stimulates autophagy, removes harmful antigens, and has the effect of enhancing anti-microbial and maintaining the homeostasis of intestinal flora. Vit D/Vit D receptor signaling pathway plays an active role in maintaining the integrity of the intestinal barrier, immune regulation and microbial regulation (6). The deficiency of Vit D/Vit D receptor can lead to impaired anti-microbial activity and increased inflammation of epithelial cells. A recent study found that a local high concentration of 1,25(OH)_2_D can promote the migration and differentiation of mouse intestinal stem cells into mature intestinal epithelial cells and enhance the repair of intestinal epithelium (16). Another prospective cohort study showed that vitamin D ≤ 35ng/ml was an independent risk factor for UC inflammation observed by endoscopy (OR 1.29, 95%CI 1.07–1.85, *p* < 0.01) and histological inflammation (OR 1.46, 95%CI 1.13–1.88, *p* = 0.005). Decreased Vit D suggests a more severe disease activity and a greater risk of future clinical recurrence and a shorter recurrence time as well (17). The increased risk of Vit D deficiency in UC patients may be associated with impaired absorption of nutrients and bile salts, dietary restrictions, and reduced exposure to light (18). Some scholars believe that serum 25(OH)D 20 ng/ml can meet the needs of at least 97.5% of the population, so serum 25(OH)D less than 20ng/ml (50 nmol/L) is defined as Vit D deficiency (19). Although the criteria for defining Vit D deficiency in previous studies were different, we did not use Vit D deficiency or not as binary variables for calculation, but direct used specific Vit D measurements for prediction, making the prediction model more accurate. As a result, our study showed a strong negative correlation between Vit D levels and the prediction model.

The acute phase of intestinal inflammation in inflammatory bowel disease was accompanied by changes in related protein synthesis. ALB and PAB, as negative acute-phase proteins, are inhibited in their synthesis, while Fbg and plasminogen involved in coagulation, as positive acute-phase proteins, are increased in their (4, 20). Weeke (21) studied 36 cases of UC (23 cases of moderate to severe UC). Results showed that the average ALB (34.6 vs. 45.7g/L) and PAB (0.17 vs. 0.24g/L) in UC patients are significantly lower than those in healthy controls (*p* < 0.01). Reductions in ALB and PAB correlated with the severity of the disease. In addition, low ALB levels at diagnosis of UC can predict the occurrence of acute severe colitis in the future (22).

Fbg is a soluble glycoprotein involved in the clotting process and the development of colitis. Animal experiments showed that Fbg induced activation of serine/threonine kinase 1 and depolymerization of microfilaments. The reduction of intercellular microfilaments promotes cell-to-cell disaggregation, destroys vascular barriers, increases colon vascular permeability, promotes inflammatory cell infiltration, and promotes the occurrence of colitis (7). A recent study found that median Fbg in patients with active UC was significantly higher than that in patients with remission UC (2.7 vs. 4.0g/L, *p* < 0.001) and was positively correlated with clinical Mayo score in UC patients (*r* = 0.529, *p* < 0.001) (23). In our study, low Vit D, ALB, PAB, and high Fbg were the risk factors for moderate to severe endoscopic activity of UC, which was consistent with the results of previous studies.

Events per variable (EPV) is the most important indicator to test the effectiveness of the model. When EPV ≥ 10, the regression coefficient is stable, and the constructed model has no risk of underestimating or overestimating (24). In this study, 45 patients with moderate to severe UC activity were included in the model, including 4 variables with EPV values greater than 10 (45/4), indicating good accuracy of the model. The dynamic nomogram made through minimally-invasive blood biomarkers had a friendly digital interface and is convenient to use, which helps clinicians to determine the severity of UC patients quickly. The model has the following advantages: the prediction model of moderate to severe endoscopic activity of UC can reduce the frequency of colonoscopy and potential risks, reduce the cost of the examination, reduce the medical burden of patients, and more in line with health economics. In addition, the model we established is based on blood biomarkers, which can be used in primary hospitals to promote UC patient management in the local community.

There are still some limitations in this study. First, as the model includes Vit D, which is affected by dietary Vit D supplementation, the prediction model may only apply to those who have not taken Vit D supplementation recently. Second, most clinical studies show a strong relationship between fecal calprotectin concentration and UC endoscopic activity, but most patients did not detect fecal calprotectin in our study, so it was not analyzed, which deserved further research in the future. Fecal calprotectin can be used to distinguish between UC in remission or active phase, but generally cannot distinguish the degree of disease activity, and the concentration of fecal calprotectin is correlated with the number of intestinal neutrophils, therefore, intestinal inflammation from other diseases can also be elevated (such as viral or bacterial infections and gastrointestinal bleeding). In addition, there may be regional variability in fecal calprotectin levels. Internationally, fecal calprotectin values of <150 μg/g is considered to reflect remission, while the Chinese patient population suggests a cut-off value of 50 μg/g is more appropriate (8). This suggests that the non-invasive biomarkers of fecal calprotectin also have certain limitations. Although not including fecal calprotectin, our prediction model provides a new monitoring method for exploring the endoscopic activities of UC. Third, recent studies have also pointed out the role of artificial intelligence in UC disease activity and surveillance, and therapy monitoring (25). It is possible to further study whether there is a superposition effect between non-invasive indicators and artificial intelligence, to guide the management of UC patients. Furthermore, our model could only distinguish UC patients with moderate to severe active from those with mild active or remission. Last, there is no external validation of the model. In the future, further external data are needed to verify the reliability of the model.

## Data availability statement

The raw data supporting the conclusions of this article will be made available by the authors, without undue reservation.

## Ethics statement

The studies involving human participants were reviewed and approved by Ethics Committee of Shanghai Oriental Hospital. Written informed consent from the participants or their legal guardian/next of kin was not required to participate in this study in accordance with the national legislation and the institutional requirements.

## Author contributions

FL and XZ: conceptualization. FL, XZ, XL, and YL: writing—review and editing. XL: data curation and analysis. XL, XZ, and ZT: methodology. XL: writing—original draft. All authors contributed to the article and approved the submitted version.

## Footnotes

1
https://mydynnom.shinyapps.io/vitD_Liu/
