# Supplementary figures and images for: Risk prediction model based on blood biomarkers for predicting moderate to severe endoscopic activity in patients with ulcerative colitis
Source: Front Med (Lausanne). 2023 Feb 21;10:1101237. doi: 10.3389/fmed.2023.1101237 (PMC9989155; doi:10.3389/fmed.2023.1101237)

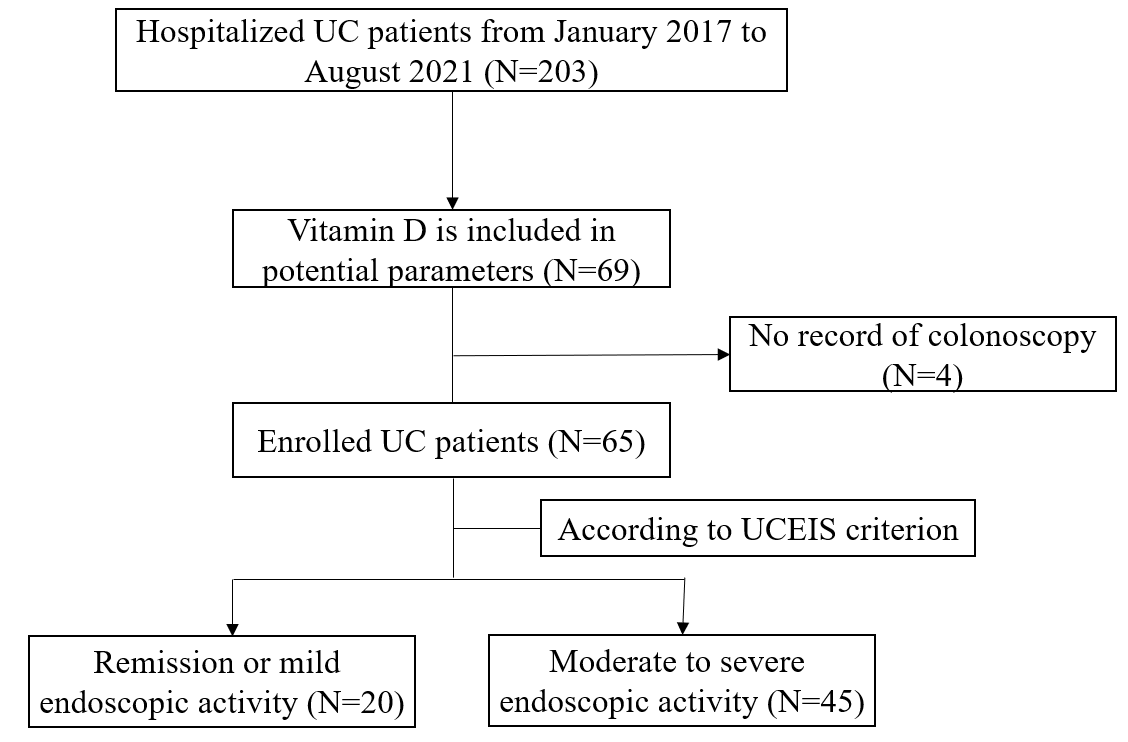

Supplement: Supplementary file 1 [file Image_1.TIF]
